# The (Ir)relevance of Group Size in Health Care Priority Setting: A Reply to Juth

**DOI:** 10.1007/s10728-016-0333-3

**Published:** 2016-10-12

**Authors:** Lars Sandman, Erik Gustavsson

**Affiliations:** 1 National Centre for Priority Setting in Health-Care, Linköping University, 581 83 Linköping, Sweden; 2 Academy for Care, Work-Life and Welfare, University of Borås, 510 90 Borås, Sweden; 3 Division of Philosophy, Department of Culture and Communication, Linköping University, Linköping, Sweden

**Keywords:** Priority setting, Orphan drugs, Rare diseases, Equality, Formal equality, Justice, Cost-effectiveness

## Abstract

How to handle orphan drugs for rare diseases is a pressing problem in current health-care. Due to the group size of patients affecting the cost of treatment, they risk being disadvantaged in relation to existing cost-effectiveness thresholds. In an article by Niklas Juth it has been argued that it is irrelevant to take indirectly operative factors like group size into account since such a compensation would risk discounting the use of cost, a relevant factor, altogether. In this article we analyze Juth’s argument and observe that we already do compensate for indirectly operative factors, both outside and within cost-effectiveness evaluations, for formal equality reasons. Based on this we argue that we have reason to set cost-effectiveness thresholds to integrate equity concerns also including formal equality considerations. We find no reason not to compensate for group size to the extent we already compensate for other factors. Moreover, groups size implying a systematic disadvantage also on a global scale, i.e. taking different aspects of the health condition of patients suffering from rare diseases into account, will provide strong reason for why group size is indeed relevant to compensate for (if anything).

## Introduction

In a market economy where pharmaceuticals are developed and sold for profit, patients suffering from rare diseases risk being systematically disadvantaged in getting access to treatment since they belong to a numerically small group. First, treatment becomes more expensive per patient due to the cost of development of drugs together with profit margins being distributed to fewer persons in small groups. Moreover, as the cost of developing a drug is approximately in the same cost range regardless of the size of the group [1] this tends to lead to worse cost-effectiveness than for other pharmaceuticals.^1^ Second, health care systems apply the same cost-effectiveness thresholds for pharmaceuticals for rare and common diseases (i.e. for orphan and non-orphan drugs^2^) as is suggested by some authors [12]. Recent public discussion shows that this is a real problem in current health care practice [4].

These two points *explain* how patients suffering from rare diseases may become disadvantaged; however, the focus of this article is the *ethical* issue of whether there is something unjust in being disadvantaged by the fact that one belongs to a numerically small group. The principled question is whether group size is a relevant factor that deserves compensation by accepting worse cost-effectiveness for orphan drugs (than for non-orphan drugs).^3^

Carlsson and colleagues have argued, drawing on a principle of formal equality, that group size is a factor that deserves compensation to some extent (given that certain other conditions are fulfilled) [2, 3, 9].^4^ Juth [11] has recently questioned this and other arguments for compensating patients suffering from rare diseases in this journal. Juth argues that the argument concerning (what he calls) irrelevance of group size fails. In this article we will analyse Juth’s argument and argue that since health care systems already compensate for the consequences of a number of factors for reasons of *formal equality* it remains unclear why we should not compensate for the consequences of group size for similar reasons. Rather, the burden of proof seems to lie on sceptics about such compensation to either show why there are relevant differences between the former and the latter, or relate to them in the same way. In fact, Juth’s own proviso about making exceptions for manmade injustices seems to refer to the very same type of formal equality principle. We conclude that a principle of formal equality should be one reason for allowing cost-effectiveness thresholds to be adjusted in a health-care system.

## Setting the Scene and the Argument About Compensating for Group Size

The argument outlined in Carlsson and colleagues in favor of compensating for the consequences of group size has been formulated to handle orphan drugs within the Swedish health-care system. For simplicity reasons we shall assume that the discussion takes place in a similar welfare-based context in this paper. In the Swedish context there is an ethical platform for priority setting. The platform consists of three principles: (1) a principle of human dignity, prescribing that people should be equally treated despite personal characteristics or standing in society (i.e. expressing a formal equality principle); (2) a needs-solidarity principle, prescribing that the health-care system should provide equal access to care and strive for an equal outcome when it comes to health and quality of life by taking patient need into account; and (3) a cost-effectiveness principle, prescribing that the health-care system should strive for a reasonable relationship between cost and effect. These principles have a rank ordering implying that the greater the patient need is, the higher cost-effectiveness threshold we could accept [17]. Similar sets of norms concerning patient need, cost-effectiveness and formal equality are accepted by a number of health-care systems, and therefore the argument about compensating for the consequences of group size could be generally valid (if sound) [10].

Carlsson and colleagues argue that since the size of the group to which one happens to belong is a morally irrelevant factor (following a formal equality principle), and given that we are concerned with a severe disease we should accept a higher cost-effectiveness threshold than what is normally accepted (all else being equal). Note that this argument does not imply that group size *per se* is what matters, but rather that formal equality requires us to compensate for the negative equity effects of group size. However, this does not exclude other ways for the health-care system to affect the cost of treatment [3].

## Juth’s Argument Against Compensating for the Consequences of Group Size

Juth claims that the argument for compensating for the consequences of group size fails to distinguish between *directly* and *indirectly operative factors* for priority setting, where the cost of treatment is an example of the former and group size is an example of the latter. He further claims that while cost is relevant for priority setting since it directly affects patients’ access to health care, group size is an irrelevant factor since it only indirectly affects access.^5^ He argues that besides group size there is a large number of other indirectly operating factors in relation to cost such as previous funding of research, earlier scientific endeavours, present scientific development, biological features of the patients (genetics, metabolism etc.).^6^ Juth acknowledges that most of these factors (including group size) are morally irrelevant *per se.* He then argues that discounting or compensating for the effects of all of these morally irrelevant factors would be tantamount to discounting cost altogether, i.e. it would be “unduly restrictive” not to allow indirect but irrelevant factors to influence direct and relevant factors. He therefore concludes that compensating for the effects of group size cannot be supported by reference to the moral irrelevance of group size.

Still, in the end he admits that there are indirectly operative factors that should be discounted or compensated for, exemplifying with what he refers to as manmade injustices [11]. Juth gives the following example: “…[if] demanding the ‘genetically inferior’ to cover a larger portion of the costs for their medications themselves, this could be just cause for adjusting cost (or preferably changing the institutional arrangements responsible for the injustice). However, none of the mentioned factors are of this kind, including rarity of disease” [11].

Since this is the only remark Juth makes about manmade injustices, we make the following interpretation of what he means. Requiring the “genetically inferior” to carry a greater cost for their health care than what is normally required from patients within the health-care system in question seems like an example of unwarranted discrimination since it involves treating this group differently than other groups with similar problems. However, it lessens the opportunity cost for other patient groups for their treatment. In such a system Juth would accept that society adjusted the cost, i.e. that society subsidized the cost or simply changed the discriminatory practice to alleviate the injustice. Furthermore, Juth accepts that cost-effectiveness should be adjusted for in relation to the degree of patient need or severity of the disease [11]. Hence, he accepts that patients are compensated for directly operating factors when it comes to cost and cost-effectiveness—a commonly accepted equity limitation of cost-effectiveness considerations.

## Manmade Injustice Following Juth

Is Juth correct in concluding that group size is not the kind of *manmade* injustices he leaves an opening to compensate for? Obviously, group size per se is not (normally)^7^ a manmade injustice but simply a brute fact. However, the same goes for being “genetically inferior” in Juth’s own example, whether this relates to racial features or implies having a genetic condition. What is portrayed (and rightly so) as a manmade injustice in Juth’s example, are the institutional arrangements concerning how the “genetically inferior” are valued and treated within the health-care system.

The most reasonable interpretation of why this institutional arrangement is unjust is that one group (the genetically inferior) is treated differently than other groups in society, despite the fact that there are no morally relevant differences between the two (in terms of aspects like severity of condition, effect of treatment and perhaps also cost-effectiveness of treatment etc.). The reason for the injustice seems to be a discriminatory attitude in society about the value of these people, that they are “inferior” in some way. In essence, Juth is referring to a formal equality principle when he makes his claim about the “genetically inferior”.

The lack of treatment for persons with rare diseases is *also* due to institutional arrangements, but not normally due to a discriminatory attitude in people. People with rare diseases are not viewed as inferior to other people; the problem is rather that their treatment is less cost-effective. Does this imply that a formal equality principle does not apply, since there are no discriminatory attitudes at play? In other words, if there are different treatments for different groups as a result of institutional arrangements, based on morally irrelevant aspects, but not as a result of discriminatory *attitudes*, should we not apply a formal equality principle? Or does a formal equality principle have a wider scope that requires us to consider compensating (at least to some extent) for the effects of all irrelevant differences between groups within our institutional arrangements? These questions will be explored later in this article. First we will have a look at the institutional arrangements in question, i.e. the practice of cost-effectiveness thresholds.

## Institutional Arrangements Concerning Cost-Effectiveness Thresholds

There is a large number of institutional arrangements that in the end result in the situation where patients do not get access to orphan drugs. One such arrangement is organizing pharmaceutical development and distribution in a market economy system, and setting prices to achieve certain profit margins. We could of course put regulations on the pharmaceutical market, which would affect profit margins. However, within a value based pricing system, the health-care system can (at least in principle) indirectly affect profit margins by not accepting too high margins. Thus, the problem with group size seems to remain despite such regulation. So let us ignore that for now, assuming “reasonable” profit margins.^8^ A second institutional arrangement is to use health technology assessments, including cost-effectiveness assessments in relation to cost-effectiveness thresholds, as a basis for decision-making about orphan drugs. However, which cost-effectiveness thresholds are accepted differs between countries and over time [7]. For example, in Sweden there has been a gradual shift from accepting upper thresholds in the range of 50,000–100,000 €/QALY to accepting thresholds in the range of 100,000–130,000 €/QALY. Now, in the health-economics literature we find theoretical approaches to how cost-effectiveness thresholds should be determined [7]. For a society with the objective of health-maximisation, the threshold should be decided by taking productivity of interventions and the health-care budget into account. This is done by consecutively choosing the most cost-effective intervention from all available interventions and including it in the health-care system until the budget limit is reached. The cost-effectiveness ratio of the least cost-effective intervention included into the bundle will then determine the cost-effectiveness threshold. This implies that all interventions not included will be less cost-effective than the threshold. If a new intervention is introduced, it should only be included into the bundle if it is more cost-effective than the threshold and then replace the least cost-effective intervention. The size of the health-care budget is supposed to reflect people’s willingness to pay for health and is related to the per capita GDP of the society.

In relation to this theoretical model of how to arrive at thresholds it is important to note a few things. First, actual cost-effectiveness thresholds in different societies do not seem to reflect the theoretical model, as the Swedish change in thresholds indicates [6]. Second, the model misses out on something that is accepted by most health-care systems: cost-effectiveness should be balanced by equity concerns. Third, the size of the health-care budget is the result of political decision-making and there is no *given* relationship between this budget and people’s preferences or the per capita GDP. Against this background it is clear that the thresholds will depend on a number of (more or less explicit) value judgements within society which are materialised in the institutional arrangements concerning thresholds [15].

Let us now move on to how cost-effectiveness assessment and the thresholds they relate to are balanced by equity concerns. A commonly accepted equity concern in many health-care systems, as we have indicated in the above, is degree of patient need or severity of disease. The more severe a disease is (i.e. the greater the patients’ needs are), the lower the cost-effectiveness ratio we can accept. In other words, the more severe a disease is, the higher the cost-effectiveness threshold should be—implying that there are different cost-effectiveness thresholds (explicitly or implicitly) for different levels of severity of disease (see e.g. [16]). This line of reasoning would lead us to accept different cost-effectiveness levels when comparing *severe* and *mild* rare diseases (as when we compare severe and mild common diseases). However, it does not give us reason to accept different cost-effectiveness levels between severe *rare* and *common* diseases. So, let us therefore return to the equity concern referred to above, i.e. formal equality concerns, and explore if that could motivate such differences in cost-effectiveness thresholds.

## Formal Equality Concerns in Health-Care Practice

Looking at current health-care practices in welfare states it seems as if we are already compensating for the effects of a number of indirectly operating factors, by reference to a formal equality principle—even if not the result of discriminatory attitudes per se. Consider the following examples.

- Even though it is more difficult to interpret the signs of myocardial infarction in women than in men, we find it reasonable for formal equality reasons to spend the extra resources on doing so, e.g. by using specific tests to better detect infarction [14, 19]. It this case we compensate in order to ameliorate the indirectly operating factor of biological differences between women and men.
- Even though administering drugs for cardiac failure for persons with severe dementia requires extra resources in terms of the time and effort of health-care personnel (due to patients’ lack of cognitive abilities to remember to take the drugs etc.), we find it reasonable from an formal equality perspective to spend those extra resources. In this case we compensate for the indirectly operating factor of cognitive differences between persons with and without dementia.
- Even though a different language background makes it more difficult to assess a patient’s problem and treat them, we find it reasonable to spend the extra resources overcoming this problem by using an interpreter. In this case we compensate in order to mitigate the indirectly operating factor of different language backgrounds.

The underlying rationale for accepting a higher cost for the patients in the examples above is a formal equality principle, which ideally requires that different groups with equal needs have equal treatment options. That is, to the extent that two patients have similar conditions, with similar severity of disease and similar potential effect of treatment, but the above exemplified indirectly operative factors make diagnosis or treatment more costly for one of them, we have a strong intuition that we should compensate for the difference in order to live up to the formal equality principle. In the examples above we compensate persons for the potential health or health-care effects of belonging to the group of women, to a group lacking cognitive abilities or to the group with a different language background than the majority population.

Group size is on par with factors like biological differences, language backgrounds etc. in resulting in that the treatment to the group to which the factor applies becomes more costly. However, there are a number of possibly relevant differences between these different factors. First, the extra cost associated with fulfilling the equal treatment principle might differ, i.e. the extra cost of a test for myocardial infarction (approximately 25 €, [18]), the cost of health-personnel administering drugs or the cost of an interpreter does not match the extra per patient cost of orphan drugs. Second, the extent to which it is possible to directly affect the underlying factor calling for compensation might also differ. Third, the extent to which the specific differentiating aspect is part of a more global disadvantage for the group might differ.

First, it is difficult to see how the fact that the indirectly operating factors differ in cost would constitute a *principled* difference since they all have an opportunity cost. On the other hand, the larger the opportunity cost the more motivated we should be to find other and less costly ways to change the situation in order to strive towards formal equality.

Second, formal equality have motivated the development of diagnostic tests to better detect myocardial infarction in women and over time the cost of these might sink. It might not be as easy to treat people with another language background without extra cost for diagnosis and treatment or administering drugs to people with cognitive failure. Perhaps computer-based translation programs can reduce the cost for translation somewhat and maybe robot development can have similar effects for persons with dementia. However, group size will (given the presuppositions of this article) necessarily imply an increased cost of treatment per patient and therefore imply an inescapable disadvantage (even if the costs are somewhat adjusted).

A third relevant aspect is the extent to which these different indirect operative factors systematically disadvantage a specific patient group at a more global level. The problem of diagnosing women with myocardial infarction systematically disadvantages women. Likewise when it comes to persons with dementia. On the other hand, people suffering from heart-related diseases tend to be advantaged in being part of patient groups with access to a large number of effective and cost-effective treatments. Hence, on a more global scale these women might not be very disadvantaged as a patient group. Not being a native language speaker may be associated with other socioeconomic disadvantages in society, but from a health-care perspective it can obviously disadvantage people from all kinds of patient groups. This implies that depending on which patient group they belong to, they might be otherwise advantaged. Hence, on a more global scale, specific indirectly operative factors might be more or less serious in systematically disadvantaging a patient group. Indirectly operative factors that add to the disadvantage of already disadvantaged groups or are related to groups where there are no compensating factors (on other health dimensions) could then be interpreted as more serious and important to address.

Group size is the core feature of rare diseases making them more costly to treat and there are generally no other features of patients’ health condition that compensate for this. On the contrary, these groups are often disadvantaged in that their conditions are chronic, start at a young age, and have a great degree of severity. Moreover, the health-care system generally lacks competence to identify and care for these diseases in a consistent way. Hence, even if group size might not be unique in this respect, there is a systematic disadvantage in belonging to a small patient group when it comes to the distribution of health care, compared to large number of other indirectly operating factors.

At this point we have tried to establish that if we are willing to accept higher cost of treatment to fulfil a formal equality principle for patients in relation to factors like gender or sex, cognitive abilities, language background etc., then we have reason to accept the same for higher costs due to group size. This is not a principled argument in itself for compensating for the effects of group size, but if one argues that we should *not* compensate for the effects of group size, one has to either accept that we should stop compensating for the effects of these other factors or explain why group size is different from these factors. As it turns out, we seem to have strong, or even stronger, reasons to compensate for the effects of group size since it results in a more systematic and inescapable disadvantage than some of the other indirect operative factors we already do compensate for. The examples mentioned earlier in this section also show that we do apply a formal equality principle beyond the cases resulting from discriminatory *attitudes*. To take this argument a step further we will now argue that formal equality concerns are already, indirectly and directly, a part of cost-effectiveness assessments—i.e. part of the very institutional arrangements we are considering.

## Formal Equality Concerns in Cost-Effectiveness Assessment

In health-economical evaluations relevant factors are not differentiated on an individual level and not even relevant differences on sub-group level are always taken into account. It is rather the median or medium cost of transportation, or administration of the drug etc. for the group that is brought into the evaluation [8]. In doing so, some patients are compensated for indirectly operative factors that would influence the cost of treatment in an unfavourable direction and other patients would have an even more favourable cost-effectiveness ratio for their treatment if the groups were differentiated. This might be done with reference to the impracticality of differentiating between sub-groups. However, we also find examples where this is done explicitly due to formal equality concerns, e.g. when the income levels of men and women are not differentiated in taking productivity into account (as reflected in actual statistics of the society) but a median or medium income taken over both sexes is used. This is, for example, the stance adopted by the Swedish Dental- and Pharmaceutical Agency.^9^

Once again, to enable men and women to have access to similar treatments for similar health problems, formal equality concerns set limits to how cost-effectiveness is assessed and thereby how it is allowed to enter the picture when deciding whether to fund treatments or not. Could it be argued, following Juth, that the kind of compensation allowed by the Swedish Dental- and Pharmaceutical Agency and in the above cases of myocardial infarction or interpreter use are simply unwarranted cases of compensating for the effects of indirect operative factors? Yes, at least in cases where it will have us accept treatment beyond generally accepted cost-effectiveness thresholds for diseases of corresponding severity. Such a hard-nosed stance would seem to have the following implications.

First, a formal equality principle would be invoked mostly for “symbolic” reasons. Let us explain. Assume we have a cost-effectiveness threshold of 100,000 €/QALY for severe diseases and we fund anything that has a cost-effectiveness ratio below this threshold. The extra cost of diagnosing women with myocardial infarction, even if resulting in a higher cost per QALY than diagnosing men, still keeps the cost-effectiveness ratio below the accepted threshold. Hence, when we accept to pay extra to provide women with similar treatment as men we need not refer to a formal equality principle. Reference to such a principle would not actually make a difference to whether treatment get funded or not. On the other hand, in cases where treatments for men and women, or for different language groups, would end up on different sides of the threshold, none of the treatments over the threshold would get funded. Instead, we should accept unequal treatment. Second, in order to guarantee that we do not compensate for the effects of indirectly operative factors in an unwarranted way we should distinguish between different sub-groups (perhaps even down to the individual level) in our cost-effectiveness assessments—at least in cases when treatment is close to the threshold (which many new treatments seem to be). This would be a rather impractical approach making it more difficult and time-consuming to perform cost-effectiveness assessment. If, on the other hand, it is argued that we should accept median levels of different factors in order to avoid impracticality—but not accept considerations based on formal equality concerns—practical considerations are allowed to trump ethical concerns, despite ethical concerns having a strong standing in a large number of health care systems [10].

In his argument, Juth seems to accept that there are cost-effectiveness thresholds against which to measure different treatments. We have relied on that assumption also in this paper. Above we found that setting a cost-effectiveness threshold is the result of a number of value judgements, including how to balance health-maximisation against equity concerns [7]. Referring to theoretical models of how to arrive at cost-effectiveness thresholds seems to beg the question whether, and if so, to what extent different equity concerns should affect these thresholds or not. Referring to existing cost-effectiveness thresholds is even shakier, since they seem to be developed in a more organic and less transparent way, perhaps even including a number of implicit formal equality concerns. Hence, it is difficult to see why we should accept the use of such thresholds to evaluate when we can accept higher cost for small groups and when we cannot. In the end it boils down to whether these thresholds should be set also with formal equality concerns in mind.

At the same time, accepting that we should adjust cost-effectiveness thresholds because of formal equality concerns comes at a price, or rather, an opportunity cost. That is, when we, following the principle of need, accept a higher cost for benefits accruing to the worse off, we end up spending more resources per health unit for people suffering from rare disease, which leads to less net health produced by the system. In systems where treatments of severe diseases are allowed to have a cost in overall health, acceptance of formal equality concerns with the implication of higher cost-effectiveness threshold related to group size will add to this effect. So, how much should formal equality concerns be allowed to cost?

If one accepts our line of reasoning one should also accept, at the very minimum, that formal equality concerns should be taken into consideration ceteris paribus. Now, the ceteris paribus clause implies that the concern for formal equality kicks in when comparing treatments for conditions of similar severity and similar effects—but where irrelevant group differences causes unequal access due to cost or some other factor (that could be compensated for by accepting a worse cost-effectiveness). In contrast to Juth we do claim that cost is not a directly operative factor, but rather a limiting factor.

Note that the ceteris paribus role for formal equality is a minimal implication of our argument. A more principled argument for how much more, if at all, the formal equality principle should be allowed to cost will have to, due to reasons of space, be explored elsewhere. It suffices to say that given the extent to which we already allow formal equality concerns to affect the use of resources in health care, it seems we are willing to go beyond the minimal level. At what point the buck stops will primarily be dependent on the extent to which resources are scarce within the health-care system in question and whether the opportunity cost can be distributed in a fair way.

## Conclusions

In this article we have analysed an argument brought forth by Niklas Juth regarding whether we should allow orphan drugs to be funded despite lower cost-effectiveness due to the effect of group size (an indirectly operative factor). Our analysis arrives at the following conclusions.

1. In making exceptions for manmade injustices, Juth makes a reference to a (at least limited) formal equality principle to allow for compensation for the effects of indirectly operative factors.
2. Looking at the health-care system, we find a number of examples of established compensation schemes for indirectly operative factors with reference to a formal equality principle. These go beyond the possibly limited version based on discriminative attitudes that Juth seems to accept. Comparing these with group size, we infer that we have as strong or even stronger reasons to compensate for the effects of group size.
3. Looking at cost-effectiveness assessment and thresholds, we find that the formal equality concerns are also allowed to implicitly or explicitly affect the assessments.

This analysis implies that formal equality concerns already have a strong standing in priority setting in health care leaving the burden of proof on those wanting to minimize the role of formal equality. If Juth argues that we should not compensate for the effects of group size, he needs to accept that we also should stop compensating for the indirectly operative factors we do compensate for today (outside as well as within cost-effectiveness assessments). Alternatively, Juth needs to explain the relevant differences between these indirect operative factors and group size that speaks to the disadvantage of the latter. Our analysis suggests, however, that in such comparison, group size seems to win the upper hand. This does not imply that we should strive towards formal equality at any cost whatsoever, i.e. that costs should be discounted altogether. Our conclusion is rather that we need to discuss which indirect operative factors should be compensated for and to what extent, and group size is a strong candidate for being such a factor.^10^

## Footnotes

1 Even if it is difficult to get a clear picture of the development cost for different drugs, it is often argued that the cost of developing a specific drug is approximately in the same cost range regardless of whether we are looking at orphan or non-orphan drugs [1]. Also, adaptations of the regulatory system and special funding for orphan drugs are in place to enable the development of such drugs and thus even out differences in development cost [3]. What will mainly affect the cost per patient of orphan drugs are hence the profit margins of the pharmaceutical company, and the size of the patient group. In a value based pricing system where the cost of treatment is supposed to reflect the value society places on the treatment, the pharmaceutical industry will somewhat adjust their profit margins to fit this value in order to get access to the market [5].

2 Henceforth we will employ the term orphan drugs in order to denote pharmaceuticals targeting rare diseases, well aware that this is sometimes used as a more technical term for drugs having a special license within a specific health-care system and also that there are distinctions between orphan and ultra-orphan drugs. However, in this principled argument, orphan drugs functions well as an overall term for pharmaceuticals for small patient groups (without exactly defining the limitations for being a small group).

3 Given that pharmaceuticals are developed by companies acting within a market economy, and that the cost for developing orphan drugs are generally the same as for any other drug, these companies could be given a number of incentives, not only to motivate them to take the risk of investing in the development of orphan drugs but also to be able to make a reasonable profit given the small number of patients. This may be one route to lower costs (and thereby better cost-effectiveness) for orphan drugs.

4 A problem with orphan drugs is that it might be difficult to provide strong evidence for reasonable effect. In this context we will ignore this problem, however it has been argued that over time it is possible to collect fairly strong evidence [12]. Moreover, accepting a higher cost-effectiveness threshold can be associated with conditions where the drug is set out unless proven effect during a certain period of time.

5 It might be questioned whether it is only the factors presented by Juth (i.e. severity of condition, effect and cost-effectiveness) that are directly operative and relevant. See for example the WHO-initiative on identifying relevant factors for priority setting apart from cost-effectiveness [13]. However, we will not explore that line of criticism but simply accept Juth’s claim about what is and what is not directly operative relevant factors.

6 Note that many of the indirectly operating factors on Juth’s list will influence whether or not there will be treatment at all for a health condition. Since such a broad understanding of indirect operative factors risk taking the discussion too far into problems of research funding and the relationship between research and actual treatment, we will concentrate on treatment already available on the market (or to the health-care system) (thereby also following Juth).

7 It could perhaps be the case that researchers through genetic engineering have produced a specific rare disease, a farfetched case that will be ignored in the following.

8 We do however realize that profit margins is the problem in relation to some orphan drugs.

9 Personal message, referring to internal working documents by Douglas Lundin, chief economist at the agency.

10 Joar Björk has made us aware of another such indirect operative factor that could provide a systematic disadvantage, having a disease that needs a biological drug. Within the current system and given the state of present medical science, biological drugs are more expensive to produce than synthetic drugs. Maybe this will change with continued development of medical science, and hence will become less of a systematic disadvantage in the future. But, until then, this may be another indirect operative factor that needs to be taken into account when assessing the degree of compensation to fulfil the equal treatment principle.
